# Evaluation of Infertility-Related Mobile Applications Using an Adapted APPLICATIONS Scoring System and a Holistic Care Framework

**DOI:** 10.3390/healthcare14203491

**Published:** 2026-10-17

**Authors:** Figen Yanik, Merlinda Alus Tokat

**Affiliations:** Department of Obstetrics and Gynecologic Nursing, Faculty of Nursing, Dokuz Eylul University, Izmir 35340, Turkey; merlinda_alus@yahoo.com

**Keywords:** infertility, mobile applications, mobile health, holistic care, patient-centered care

## Abstract

**Background/Objectives**: Mobile health applications often focus on physiological tracking while neglecting the provision of holistic infertility care. This study aimed to evaluate the technical and usability characteristics and comprehensiveness of holistic care provided by infertility-related mobile applications using the adapted APPLICATIONS Scoring System. **Methods**: This descriptive, cross-sectional study systematically searched the Apple App Store and Google Play Store at five intervals between 2021 and 2025. English and Turkish keywords were used to screen 2108 applications, with 79 meeting the inclusion criteria. The original APPLICATIONS Scoring System was adapted by combining its 13-point Technical and Usability Component with a researcher-developed 16-point Infertility-Specific Holistic Care Component, resulting in a 29-point adapted APPLICATIONS Scoring System. **Results:** Among the 79 applications analyzed, the mean score for the Holistic Care Comprehensiveness Component was 6.63 ± 3.50, and that for the Technical and Usability Component was 7.33 ± 2.07. The overall mean adapted APPLICATIONS score was 13.96 ± 4.95 (range, 7–28), with only 7.6% (*n* = 6) of applications providing comprehensive holistic care content. The physiological dimension was the most frequently represented (*n* = 65, 82.3%), whereas the psychological dimension (*n* = 11, 13.9%) was the least frequently represented. Only 39.2% of the applications referenced scientific literature or evidence-based sources. Regarding developers, the developer was unknown for 35.5% of the applications. Regarding the target audience, 48.1% of the applications were designed for couples, 44.3% for women, and 7.6% specifically for men. **Conclusions:** Mobile applications are increasingly used in infertility care; however, most lack a holistic perspective. To provide high-quality support, infertility applications should incorporate evidence-based content and professional guidance that address the multidimensional needs of both partners.

## 1. Introduction

In the contemporary digital era, smartphones have evolved from simple communication tools to essential components of the healthcare environment [1]. Mobile health (mHealth) applications play a crucial role in personal health management [2,3]. As these technologies advance, they offer opportunities to enhance health literacy, facilitate behavioral monitoring, and empower patients through self-management tools [3]. In reproductive health, mHealth applications have gained considerable attention [3,4]. They serve as digital adjuncts that support fertility awareness, treatment guidance, and patient education for individuals navigating infertility [5].

Infertility is a multidimensional experience that extends beyond reproductive disorders, affecting individuals’ psychological well-being, couples’ relationships, social well-being, and spiritual well-being [6]. Couples experiencing infertility may face significant emotional challenges, including anxiety, depression, grief, uncertainty, and loss of control, particularly during repeated and demanding fertility treatments [7]. Infertility can place substantial pressure on couples’ relationships by affecting communication between partners, emotional and physical intimacy, sexual quality of life, and shared coping processes [6,7]. Moreover, infertility-related stigma, social isolation, and cultural expectations regarding parenthood can exacerbate the physical, psychological, and social burden experienced by individuals and couples [8]. The uncertainty and challenges associated with the infertility journey can undermine individuals’ hopes, beliefs, and spiritual well-being [6]. However, these spiritual aspects may also serve as important coping resources during diagnosis and treatment. Taken together, the physiological, psychological, social, and spiritual effects of infertility are closely interrelated [6,7,8]. Therefore, infertility care should not be limited to biomedical treatment; it should include a holistic, couple-centered approach that addresses couples’ multidimensional needs throughout the infertility journey [9].

In this regard, the European Society of Human Reproduction and Embryology (ESHRE) recommends integrating psychosocial care into routine infertility services [8]. However, a significant care gap persists between the multidimensional needs of individuals and couples experiencing infertility and the supportive care services provided [9]. Geographical barriers, infertility-related stigma, financial constraints, and the time-consuming nature of clinical visits make it difficult to effectively meet psychosocial support needs [8,9]. In this context, mHealth applications may help narrow this care gap by facilitating access to information and support outside clinical settings, supporting continuity of care, and integrating infertility-related resources into individuals’ daily lives [10].

However, despite the growing role of mHealth applications in infertility care, important limitations remain regarding their scope and quality [11]. Specifically, existing applications are reported to focus predominantly on physiological aspects, such as fertility tracking, reproductive health indicators, and treatment timelines, while devoting limited attention to the psychological, social, and spiritual dimensions of infertility [12]. Additionally, the rapid proliferation of these applications has raised concerns that scientific evaluation may not have kept pace with their development, particularly regarding the evidence base and reliability of the information provided [13]. Although the number of mobile applications for infertility continues to increase, studies evaluating both their technical and usability characteristics and the extent to which they address holistic care needs remain limited [10,13]. Therefore, evaluating the technical and usability characteristics, as well as the scope of care content offered by mobile applications, is important for identifying areas for improvement and informing the selection and development of digital tools for infertility care [13].

This study aimed to evaluate the technical and usability characteristics of infertility-related mobile applications available on the Apple App Store and Google Play Store using an adapted APPLICATIONS Scoring System and examine their infertility-specific holistic care content using a researcher-developed evaluation component.

## 2. Materials and Methods

### 2.1. Study Design

This study employed a descriptive, cross-sectional design involving systematic mobile application store searches conducted at five time points between February 2021 and December 2025: February 2021, March 2022, December 2023, December 2024, and December 2025. The study focused on mobile applications designed for individuals or couples experiencing infertility or seeking conception-related support that were available through the Apple App Store or Google Play Store. The applications’ technical and usability characteristics, as well as their holistic care content, were evaluated using an adapted APPLICATIONS Scoring System.

Given the dynamic nature of the mobile application market, repeated searches were conducted to capture changes in application availability over time and determine the final set of eligible applications. Applications identified during earlier searches were rechecked at subsequent time points to determine their continued availability and functionality; however, APPLICATIONS scores were not compared longitudinally across the five assessment time points. Applications that were no longer available or functional at the final assessment in December 2025 were excluded from the final analysis. For applications whose features, content, or payment models changed during the study period, the version available at the final assessment in December 2025 was used for scoring. Thus, the final evaluation using the adapted APPLICATIONS Scoring System reflects the status of the included applications as of December 2025, with scores based on the versions available at that time rather than on versions assessed at earlier time points.

### 2.2. Application Search Strategy

Search keywords were identified based on a review of the relevant literature. The application store search strategy included the following corresponding search terms in English and Turkish: infertility (infertilite, kısırlık), fertility (fertilite, doğurganlık), in vitro fertilization/IVF (tüp bebek), sterility (sterilite), and conceive (gebe kalma). To reflect the typical end-user search experience, searches were performed on two separate smartphones: an Apple iPhone for the Apple App Store (iOS) and a Samsung Android smartphone for the Google Play Store (Android). All searches were conducted using regional settings for Turkey in the Apple App Store and Google Play Store. The country/region settings were not changed during the search process. The searches were conducted without logging into a user account to minimize potential personalization based on the account’s search history. All applications retrieved through the searches were systematically recorded and screened without applying store-ranking or search-result limits.

Duplicate records resulting from different search terms and applications identified across multiple time points were consolidated so that each application was represented only once in the final dataset. Applications available in both stores were considered a single application (*n* = 1) and evaluated once rather than separately for each platform. Cross-platform duplicates were identified by comparing the application name, developer, and core functionality. Availability on both platforms was recorded under the “inter-platform compatibility” criterion of the adapted APPLICATIONS Scoring System.

### 2.3. Application Selection Process

The application selection process comprised four sequential phases. (1) Identification: Potentially relevant mobile applications were identified in the Apple App Store and Google Play Store using the predefined bilingual search terms. (2) Screening: Duplicate records were removed, and irrelevant applications were preliminarily excluded based on store descriptions and available information. (3) Eligibility: Applications deemed potentially eligible were assessed in detail to confirm their functionality, target audience, and features, and to evaluate their content related to infertility, fertility, or conception. (4) Inclusion: Eligible applications were included in the systematic evaluation using the adapted APPLICATIONS Scoring System.

### 2.4. Application Inclusion and Exclusion Criteria

Applications were included if they met all of the following criteria: (1) provided support, features, or content related to infertility, fertility awareness, conception planning, or assisted reproductive technology (ART); (2) were accessible in English or Turkish; and (3) were functional and available on the Apple App Store or Google Play Store at the final assessment in December 2025.

Multifunctional applications, such as general women’s health or pregnancy-related applications, were included if they contained content or features specifically designed to support individuals or couples trying to conceive or experiencing infertility (e.g., predicting fertile windows, tracking ovulation, providing conception guidance, or offering fertility-related modules). Conversely, applications were excluded if they (1) focused exclusively on pregnancy or postpartum care without preconception or fertility support; (2) functioned solely as menstrual calendars or contraceptive trackers without conception-focused features; (3) lacked identifiable infertility- or fertility-related content; (4) targeted healthcare professionals only; (5) required restricted institutional or clinic-specific login credentials; or (6) had persistent technical problems that prevented systematic evaluation.

### 2.5. The Adapted APPLICATIONS Scoring System

An adapted APPLICATIONS Scoring System was used in this study to reflect the multidimensional nature of infertility and the holistic care needs associated with infertility [14]. Other established application-evaluation scoring systems serve different but complementary purposes. The Mobile Application Rating Scale (MARS) enables a multidimensional assessment of overall application quality based on engagement, functionality, aesthetics, information quality, and subjective quality, whereas ORCHA-based frameworks focus on broader areas of digital health assurance, such as data privacy, professional and clinical assurance, usability, security, and regulatory requirements [15,16]. Although MARS and ORCHA are well-established approaches for evaluating mobile health applications, they are not fully aligned with the specific aims of this study, which were to evaluate technical and usability characteristics and infertility-specific holistic care content [14,15,16]. The original and adapted APPLICATIONS Scoring Systems do not include data privacy, cybersecurity, or regulatory compliance as scoring domains.

The adapted APPLICATIONS Scoring System was considered more directly aligned with the objectives of this study because of its criteria-based structure and adaptable comprehensiveness component. The original APPLICATIONS Scoring System was developed by Chyjek et al. [14] to evaluate pregnancy-related mobile applications. It has since been adapted to align with the clinical context and objectives of the applications being evaluated for use in various areas of women’s health [17,18,19,20,21]. This adaptability allows the scoring system to accommodate the specific clinical focus of different evaluations. The criteria-based structure of the original APPLICATIONS Scoring System enables the systematic evaluation of observable technical and usability characteristics, while its adaptable comprehensiveness component allows content criteria to be tailored to the specific research question and clinical context of a study.

Accordingly, the basic structure of the original APPLICATIONS Scoring System was preserved, and the comprehensiveness component was adapted to reflect the physiological, psychological, social, and spiritual dimensions of infertility care. The adapted APPLICATIONS Scoring System used in this study consists of two main components: (1) the 13-point Technical and Usability Component, based on the original APPLICATIONS criteria, and (2) the 16-point Infertility-Specific Application Comprehensiveness Component, developed for this study to assess the physiological, psychological, social, and spiritual dimensions of infertility care. Thus, the total possible score ranges from 0 to 29. Higher scores indicate a greater number of technical and usability features and broader coverage of infertility-specific holistic care content.

#### 2.5.1. Technical and Usability Component

The Technical and Usability Component evaluates the functional infrastructure and technical design of the applications. The evaluation criteria include price, subscription options, use of scientific literature, availability of in-app purchases, connectivity features, advertisements, the presence of a text-search function, inter-platform compatibility, inclusion of images/figures and videos, special features, ease of navigation, and subjective presentation [14].

The original APPLICATIONS Scoring System primarily uses binary scoring. Eleven criteria are scored according to the presence or absence of the specified characteristic (1 or 0, respectively), thereby reducing variability among evaluators. The remaining two criteria, ease of navigation and subjective presentation, are initially rated on a five-point scale ranging from 1 (poor) to 5 (excellent). These ratings are converted into binary scores according to the original APPLICATIONS scoring rule: ratings of 3 or higher receive 1 point, whereas ratings below 3 receive 0 points [14].

Two researchers independently evaluated all applications; discrepancies in scoring were resolved through discussion until consensus was reached. Each of the 13 criteria contributed a maximum of one point, resulting in a total score ranging from 0 to 13 for the Technical and Usability Component. Table 1 provides detailed definitions and scoring rules for all criteria.

**Table 1:** Scoring criteria for the adapted APPLICATIONS Scoring System.

Component	Evaluation Criterion	Score	Scoring Rule
**Part I:** Infertility-Specific Application Comprehensiveness **(A)**			
Physiological Dimension	Infertility-related physiological and clinical content	0 or 4	0 = None of the predefined criteria are present; 4 = At least one predefined criterion is present (see Table 2)
Psychological Dimension	Psychological and emotional support content	0 or 4	0 = None of the predefined criteria are present; 4 = At least one predefined criterion is present (see Table 2)
Social Dimension	Social and interpersonal support content	0 or 4	0 = None of the predefined criteria are present; 4 = At least one predefined criterion is present (see Table 2)
Spiritual Dimension	Spiritual well-being and coping content	0 or 4	0 = None of the predefined criteria are present; 4 = At least one predefined criterion is present (see Table 2)
**Part II:** Technical and Usability Component			
Price (P)	Cost of downloading the application from the marketplace	0 or 1	0 = Paid download; 1 = Free download
Paid Subscription (P)	Mandatory subscription required to access core features	0 or 1	0 = Subscription required; 1 = No subscription required
Literature Used (L)	Inclusion of references to peer-reviewed scientific studies or clinical guidelines	0 or 1	0 = No references; 1 = Scientific references cited
In-App Purchases (I)	Monetized add-on features or paywalled content	0 or 1	0 = In-app purchases present; 1 = No in-app purchases
Connectivity (C)	Ability to function without continuous network access	0 or 1	0 = Constant internet access required; 1 = Offline functionality available
Advertisements (A)	Presence of commercial pop-ups or third-party advertising banners	0 or 1	0 = Advertisements present; 1 = Ad-free interface
Text Search Field (T)	In-app keyword search function	0 or 1	0 = Absent; 1 = Present
Inter-Platform Compatibility (I)	Availability on both the Apple App Store and Google Play Store	0 or 1	0 = Available on a single platform (iOS or Android); 1 = Available on both platforms
Visual Images/Figures (O)	Diagrams, anatomical illustrations, or educational graphics	0 or 1	0 = Absent; 1 = Present
Video Content (O)	Educational or instructional videos	0 or 1	0 = Absent; 1 = Present
Special Features (O)	Additional tools *	0 or 1	0 = Absent; 1 = At least one special feature present
Navigation Ease (N)	Ease and intuitiveness of navigating the application interface	0 or 1	0 = Likert score < 3; 1 = Likert score ≥ 3
Subjective Presentation (S)	Visual aesthetics, layout, graphic quality, and overall professional appearance	0 or 1	0 = Likert score < 3; 1 = Likert score ≥ 3
**Total**	**Part I (0–16) + Part II (0–13)**	**0–29**	**Sum of all scores**

* Special features include tutorials, customizable reminders, social media links, security/password protection, and medical disclaimers. Note: The original APPLICATIONS Scoring System consists of the 13 criteria presented in Part II (score range: 0–13). Part I (score range: 0–16) represents an infertility-specific holistic care component developed by the authors to capture the physiological, psychological, social, and spiritual dimensions of infertility care.

**Table 2:** Infertility-specific holistic care criteria used in the Application Comprehensiveness Component.

Holistic Care Dimension	Predefined Criteria	Score
Physiological Dimension	Information on infertility etiology and diagnostic assessment; infertility treatments and ART/IVF protocols; medication- and hormone-related information; lifestyle factors relevant to fertility; and reproductive health information, including ovulation, menstruation, sexual health, and gamete quality.	0 or 4
Psychological Dimension	Content addressing emotional distress and psychological well-being; stress and anxiety management; coping strategies; cognitive-behavioral approaches; standardized mental health screening; relationship-related psychological support; and access to professional psychological support.	0 or 4
Social Dimension	Features supporting peer interaction and online fertility communities; couple/dyadic communication; communication with healthcare professionals; and access to professional counseling or fertility-related guidance.	0 or 4
Spiritual Dimension	Content addressing spiritual well-being and complementary coping practices, including mindfulness, meditation, relaxation, self-compassion, yoga, breathing exercises, prayer, music, and other spiritual or complementary practices.	0 or 4

Note: Each dimension was scored dichotomously: 4 = at least one predefined criterion within the dimension was present; 0 = none of the predefined criteria were present. Intermediate scores of 1, 2, or 3 were not assigned.

#### 2.5.2. Application Comprehensiveness (A) Component

Infertility affects multiple dimensions of health, including psychological, social, and spiritual well-being [6,11]. Therefore, an infertility-specific holistic framework was incorporated into the adapted APPLICATIONS Scoring System to evaluate holistic care content beyond technical and usability characteristics.

The Application Comprehensiveness Component (A) was developed for this study to evaluate four dimensions of holistic infertility care: physiological, psychological, social, and spiritual. The criteria for each dimension were developed through a review of the relevant literature on infertility and holistic care [22,23,24,25,26,27,28,29]. Recurring care needs and areas of support identified in the literature were grouped into these four dimensions.

Physiological Dimension (4 points): This dimension assessed content related to infertility etiology, diagnostic tests, ART/IVF protocols, medication tracking, hormone values, and gamete quality [22,23].

Psychological Dimension (4 points): This dimension assessed content related to emotional distress management, coping strategies, and standardized mental health screening [24,25].

Social Dimension (4 points): This dimension assessed features or content related to peer support, couple communication, online communities, and access to professional counseling [22,23,24,25].

Spiritual Dimension (4 points): This dimension assessed content related to spiritual well-being, mindfulness, meditation, relaxation, and complementary coping practices [26,27,28,29].

Each dimension was scored dichotomously according to the presence or absence of the predefined criteria. An application received 4 points for a given dimension if at least one of the predefined criteria within that dimension was present, and 0 points if none of the criteria were present; intermediate scores of 1, 2, or 3 were not assigned. This dichotomous scoring approach assessed the presence or absence of each holistic care dimension rather than the depth or quality of the content within that dimension. The four dimensions were equally weighted at 4 points each, resulting in a maximum possible Application Comprehensiveness score of 16 points. Comprehensive holistic care content was defined *a priori* as content covering all four dimensions of holistic care (physiological, psychological, social, and spiritual); mobile applications addressing all four dimensions were classified as providing comprehensive holistic care content. This weighting approach placed greater emphasis on infertility-specific clinical and health-related content than on individual technical features in accordance with the aim of the study. Equal weighting across the four holistic dimensions ensured that no greater *a priori* importance was assigned to any single dimension.

The content evaluation criteria were reviewed for relevance and clarity by a multidisciplinary panel of three experts comprising a nursing academic, a fertility nurse, and a gynecologist. The criteria were refined based on expert feedback, and the final Application Comprehensiveness Component was established by consensus. Formal quantitative content validity assessment using the item-level Content Validity Index or scale-level Content Validity Index and psychometric validation were not performed; therefore, the Application Comprehensiveness Component represents a study-specific, criteria-based evaluation tool rather than a validated psychometric instrument.

The adapted APPLICATIONS Scoring System therefore consists of a 13-point Technical and Usability Component and a 16-point Infertility-Specific Application Comprehensiveness Component, resulting in a total score ranging from 0 to 29. Higher scores indicate a greater number of technical and usability features as well as broader coverage of infertility-specific holistic care dimensions (Table 1 and Table 2).

### 2.6. Statistical Analysis

Statistical analyses were performed using IBM SPSS Statistics for Windows, version 24.0 (IBM Corp., Armonk, NY, USA). Inter-rater reliability between the two independent evaluators was assessed using the intraclass correlation coefficient (ICC) based on a two-way mixed-effects model with absolute agreement and single measures. Categorical variables were summarized using frequencies and percentages. Overall score distributions were described using means, standard deviations, and ranges, whereas group-specific scores were summarized using medians and interquartile ranges. The Shapiro–Wilk test was used to assess the distribution of total adapted APPLICATIONS scores. Since the scores were not normally distributed (*p* < 0.001), nonparametric tests were used for group comparisons. Mann–Whitney *U* tests were used for comparisons between two independent groups, and Kruskal–Wallis *H* tests were used for comparisons involving three independent groups. Significant Kruskal-Wallis results were followed by pairwise comparisons with a Bonferroni adjustment. All statistical tests were two-sided, and a value of *p* < 0.05 was considered statistically significant.

## 3. Results

**Figure 1:**
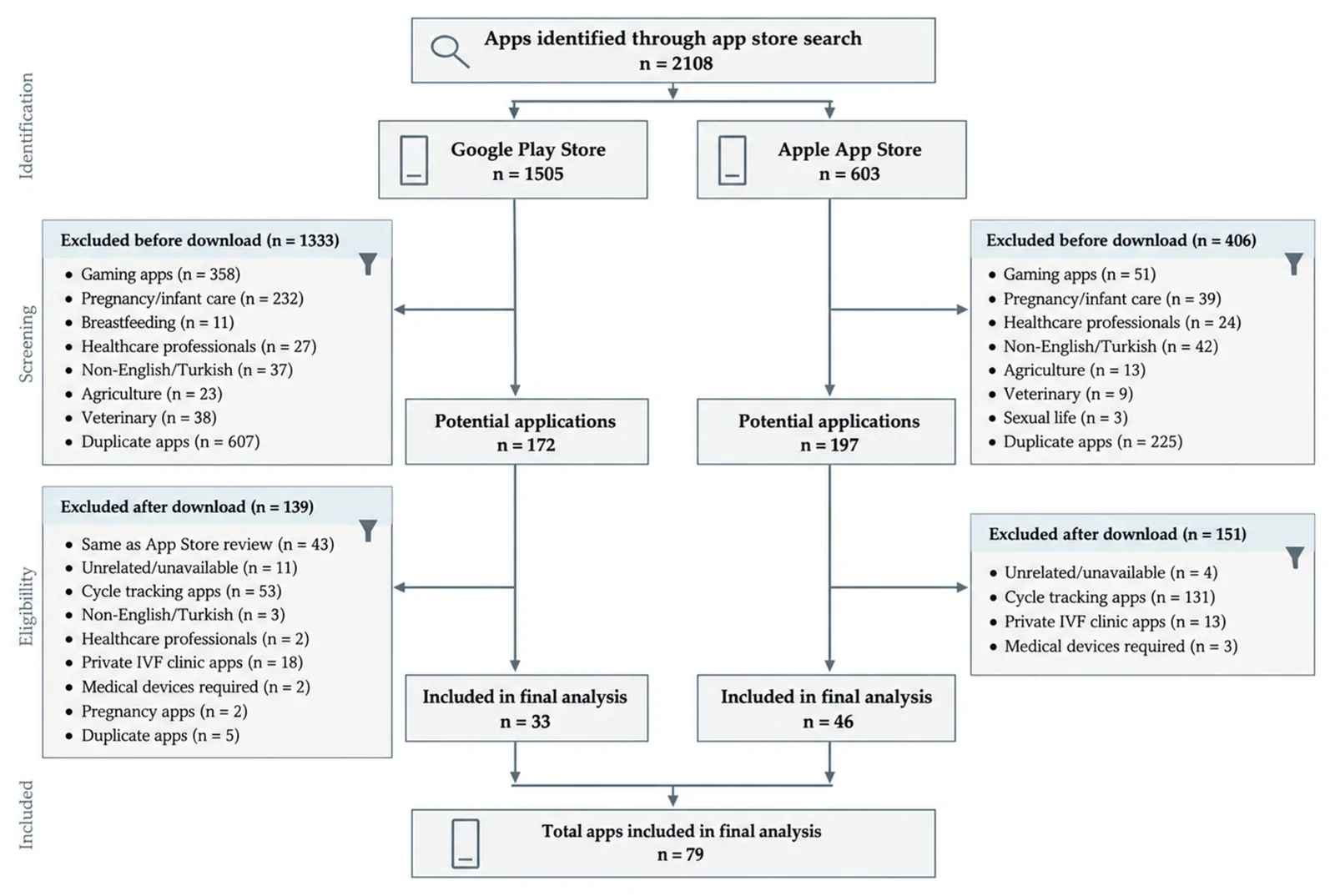
Flow diagram of the application selection process used in the study.

A total of 2108 applications were initially identified through searches on the Google Play Store (*n* = 1505) and the Apple App Store (*n* = 603). Following the screening and eligibility assessments, 33 applications from the Google Play Store and 46 applications from the Apple App Store met the inclusion criteria. Ultimately, 79 applications were included in the final analysis (see Figure 1).

Inter-rater reliability between the two evaluators was excellent (ICC = 0.991, 95% CI: 0.986–0.994, *p* < 0.001).

The mean total score on the adapted APPLICATIONS Scoring System was 13.96 ± 4.95 (range, 7–28). Nine applications (11.4%) received a total score of 20 or higher: Glow-Fertility & Ovulation, Fertility Circle: Support Hub, Enhanced Fertility, Ovia Fertility & Cycle Tracker, Tilly: Fertility & IVF Support, FertilityNow, 1in4, Sperm Count Advice, and Mindful IVF: Meditate & Relax. Table 3 presents detailed scores for each application across the evaluated criteria.

The mean score for the Application Comprehensiveness Component was 6.63 ± 3.50 (range, 4–16), while the mean score for the Technical and Usability Component was 7.33 ± 2.07 (range, 3–12). Six out of the 79 applications (7.6%) addressed all four holistic care dimensions and therefore met the *a priori* definition of comprehensive holistic care content. These applications included Glow-Fertility & Ovulation, Fertility Circle: Support Hub, Ovia Fertility & Cycle Tracker, Enhanced Fertility, Tilly: Fertility & IVF Support, and FertilityNow.

Among the holistic care dimensions, the physiological dimension was the most frequently represented (*n* = 65, 82.3%), followed by the spiritual (*n* = 30, 38.0%) and social (*n* = 25, 31.6%) dimensions. The psychological dimension was the least frequently represented (*n* = 11, 13.9%).

Regarding technical and usability characteristics, 73 applications (92.4%) were available for download at no cost, 75 (94.9%) offered subscription options, 31 (39.2%) referenced scientific literature, 50 (63.3%) included in-app purchases, 19 (24.1%) included connectivity features, and 52 (65.8%) contained advertisements. Fourteen applications (17.7%) had text search functionality, and 33 (41.8%) were compatible with multiple platforms. Seventy-one applications (89.9%) included images or visual materials, 15 (19.0%) included videos, and 41 (51.9%) included special features. Additionally, 60 applications (75.9%) met the navigation criterion, and 45 (57.0%) met the presentation criterion (see Table 3).

**Figure 2:**
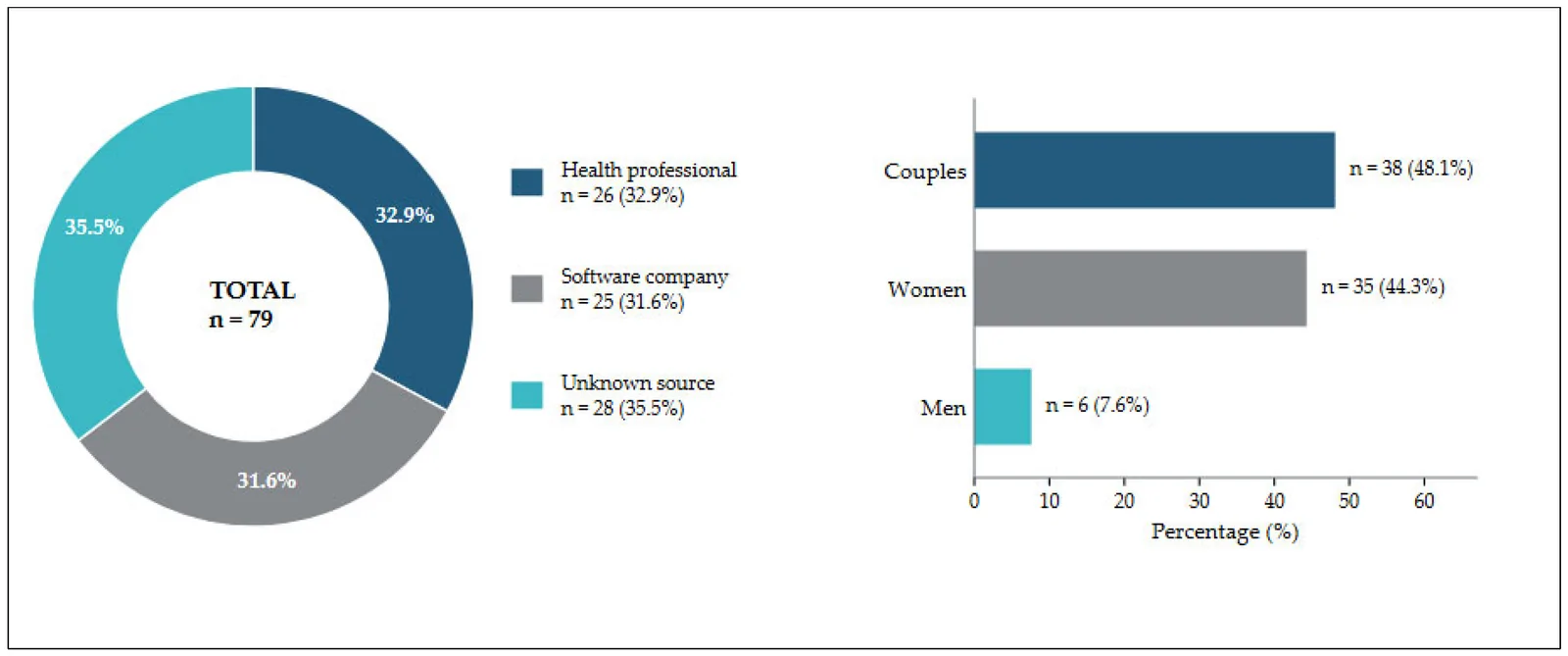
Developer type and target audience of infertility-related mobile applications (*n* = 79).

Among the 79 mobile applications included in the analysis, 26 (32.9%) were developed by healthcare professionals, 25 (31.6%) by software companies, and 28 (35.5%) had unknown developers. Regarding the target audience, 38 applications (48.1%) were designed for couples, 35 (44.3%) for women, and six (7.6%) specifically for men (see Figure 2).

When the total scores on the adapted APPLICATIONS Scoring System were compared across application characteristics, statistically significant differences were observed across platform, developer type, and target audience; however, no significant difference was found by price. Scores differed significantly across platform (*H* = 12.438, *p* = 0.002). The median score for applications available on both platforms was 16 (IQR = 8), compared with 11 (IQR = 7) for iOS-only applications and 11 (IQR = 5) for Android-only applications. Bonferroni-adjusted pairwise comparisons showed that applications available on both platforms had significantly higher scores than those available only on iOS (*p* = 0.016) or only on Android (*p* = 0.006). Significant differences were also observed across developer type (*H* = 62.645, *p* < 0.001), with median scores of 18 (IQR = 7) for applications developed by healthcare professionals, 13 (IQR = 4) for those developed by software companies, and 9 (IQR = 1) for those with unknown developers. Applications developed by healthcare professionals had significantly higher scores than those developed by software companies and those with unknown developers (*p* < 0.001 for both). Applications developed by software companies also had significantly higher scores than those with unknown developers (*p* = 0.001). Similarly, scores differed significantly across target audience (*H* = 44.251, *p* < 0.001), with median scores of 16 (IQR = 4) for applications targeting couples, 10 (IQR = 2) for those targeting women, and 11 (IQR = 4) for those targeting men. Applications targeting couples had significantly higher scores than those targeting women (*p* < 0.001), whereas the other pairwise comparisons were not significant. In contrast, no significant difference was found by price, with median scores of 13 (IQR = 7) for free applications and 12 (IQR = 9) for paid applications (*U* = 203.50, *p* = 0.773) (see Table 4).

## 4. Discussion

The findings of this study suggest that infertility-related mobile applications provide limited coverage of holistic care needs. Among the 79 applications evaluated, the mean adapted APPLICATIONS score was 13.96 ± 4.95, and only six applications (7.6%) addressed all four dimensions of holistic care, meeting the *a priori* definition of comprehensive holistic care content. Despite the large number of applications identified through infertility-related searches, only a small proportion provided support across all dimensions of holistic infertility care. Previous studies have similarly highlighted limitations in the relevance, comprehensiveness, and evidence base of mobile health applications related to infertility and reproductive health [30]. Taken together, these findings emphasize the need for infertility-related applications that integrate evidence-based information with support that addresses the multidimensional needs of individuals and couples experiencing infertility [30,31].

Further examination of the search results revealed a discrepancy between the infertility-specific search terms and the applications retrieved from the application stores [32]. Many of the identified applications were unrelated to infertility care, such as those focused on veterinary contexts or basic menstrual cycle tracking [33]. This suggests that individuals seeking infertility-specific digital support may need to navigate numerous applications before finding relevant resources [30,32]. Therefore, greater attention should be directed toward ensuring the relevance and comprehensiveness of the information provided by mobile applications intended to support individuals and couples experiencing infertility [30,31,32,33].

When the applications were examined from a holistic care perspective, the physiological dimension was the most frequently represented (82.3%), whereas psychological content was substantially less common (13.9%). This imbalance is clinically relevant because repeated treatment cycles, invasive procedures, and uncertainty regarding treatment outcomes can contribute to psychological distress, including anxiety, depressive symptoms, and feelings of loss among individuals experiencing infertility [34]. Despite these recognized psychosocial challenges, only a small proportion of the evaluated applications included structured psychological support. The ESHRE guidelines recommend evaluating emotional well-being throughout infertility treatment and using validated screening tools specific to infertility to identify individuals at risk of psychosocial difficulties [8]. In this context, mobile applications may complement psychosocial care by providing access to evidence-based coping resources and facilitating referrals to professional support when needed, rather than replacing clinical assessment [35]. The limited representation of these features indicates a clear opportunity for improvement in the digital support available to individuals experiencing infertility.

In line with this perspective, mHealth technologies may help address psychological needs throughout fertility care [36]. Meyers and Domar highlighted the potential of fertility-related digital interventions that integrate clinical information with emotional support features to facilitate mood monitoring and support the development of coping strategies during treatment [37]. This evidence further underscores the need for infertility-related mobile applications to extend beyond physiological tracking and incorporate evidence-based psychosocial support that addresses the multidimensional needs of individuals and couples experiencing infertility [36,37].

Only 39.2% of the evaluated applications referenced scientific literature, suggesting that the use of scientific sources was limited among the infertility-related mobile applications evaluated [38]. Similar concerns have been reported in previous studies. Zwingerman et al. found that 58.3% of infertility-related applications lacked references to scientific literature and also identified inaccuracies in the information provided [39]. These findings are consistent with the limited use of scientific references observed in the present study and highlight the importance of strengthening the scientific basis of digital resources for infertility care [38,39]. To support the evaluation of digital health technologies, organizations such as the National Institute for Health and Care Excellence (NICE) have developed evidence standards that consider the evidence required according to the intended purpose and level of risk [40]. Such frameworks may assist healthcare professionals in critically evaluating digital resources before recommending them to individuals experiencing infertility.

This evaluation highlights that the technical features and usability of fertility-related mobile health applications should be distinguished from clinical quality [41]. Although an application may perform well in terms of navigation, visual presentation, and functionality, the health information it provides may be limited, inadequately cited, or insufficiently evidence-based [42]. Therefore, professional design and high usability should not be interpreted as indicators of clinical reliability. The scientific evidence supporting the content and its alignment with current reproductive health guidelines should also be considered when evaluating such applications [41,42].

This distinction directly informed the adaptation of the APPLICATIONS Scoring System in this study. While the scoring structure of the original Technical and Usability Component was retained, the adapted system added a 16-point Infertility-Specific Application Comprehensiveness Component to reflect the holistic focus of the study. This adaptation allowed the assessment to incorporate the physiological, psychological, social, and spiritual dimensions of infertility care. Importantly, higher total scores obtained using the adapted system should not be interpreted as evidence of clinical validity, patient safety, diagnostic accuracy, or effectiveness in improving reproductive outcomes. Rather, they reflect the presence of a greater number of technical and usability features and broader coverage of infertility-specific holistic care dimensions.

The widespread use of mobile applications raises concerns about users’ potential exposure to inaccurate or insufficiently supported health information [39]. Although infertility-related applications can provide useful support during infertility care, inaccurate or misleading information may influence users’ health-related decisions [43]. Collaboration between application developers and healthcare professionals is therefore important to strengthen the scientific basis and clinical relevance of digital health content, while healthcare professionals can guide patients toward appropriate digital resources [39,40]. The adapted APPLICATIONS Scoring System provides a structured approach to evaluating technical and usability characteristics together with the comprehensiveness of infertility-specific holistic care content. However, because this system does not assess clinical validity, safety, or the accuracy of health information, these aspects require separate evaluation when considering applications for use in infertility care. Nonetheless, the limited representation of comprehensive holistic care content and scientific references highlights areas in which infertility-related mobile applications could be strengthened.

Another notable finding was that only 41.8% of the evaluated applications (*n* = 33) were available on both the Apple App Store and Google Play Store. These applications had significantly higher adapted APPLICATIONS scores than those available exclusively on iOS or Android. Limited availability across operating systems may restrict users’ access to digital resources depending on the operating system used on their devices [41]. Expanding access across platforms, particularly for applications providing comprehensive infertility-specific support, could improve accessibility for individuals and couples experiencing infertility [44,45].

Another important finding concerned the source of application development. Only 32.9% of the evaluated applications were developed by healthcare professionals, whereas 35.5% had unknown developers. Applications developed by healthcare professionals had higher adapted APPLICATIONS scores than those developed by software companies or those with unknown developers. Although developer type alone does not establish the accuracy or clinical reliability of an application, the high proportion of unidentified developers raises concerns about transparency and accountability in infertility-related digital health resources [42]. Healthcare professionals should therefore consider both the source of an application and the scientific basis of its content when guiding individuals and couples toward digital resources [43].

In contrast, no statistically significant difference in adapted APPLICATIONS scores was observed between free and paid applications. This finding suggests that price was not significantly associated with the technical and usability characteristics or the infertility-specific holistic care comprehensiveness assessed in this study. Therefore, paying for an application should not be assumed to provide broader holistic care content or a greater number of technical and usability features [41,43].

Mobile applications also appear to provide limited representation of the relational and couple-focused dimensions of infertility [46]. Infertility is not solely an individual experience; it affects both partners, influencing communication, intimacy, shared decision-making, and coping within the relationship [47]. However, digital infertility support has predominantly focused on women, which may limit opportunities to involve both partners throughout the process [46,47]. Greater attention to partner involvement and dyadic coping may therefore help digital interventions better address the relational dimensions of infertility.

This women-centered emphasis is reflected in the limited representation of men in infertility-related digital health content [48]. In the present study, 44.3% (*n* = 35) of the applications targeted women, whereas only 7.6% (*n* = 6) specifically targeted men. This difference is noteworthy because male factors contribute to a substantial proportion of infertility cases, either alone or in combination with female factors [48]. Previous research suggests that stigma surrounding male infertility and perceptions of infertility as a threat to masculinity can discourage men from seeking information or professional support [48,49]. Including men more actively in digital infertility support could improve access to evidence-based information on male reproductive health and modifiable lifestyle factors [49]. It could also facilitate partner involvement, shared decision-making, and dyadic coping throughout the infertility journey [48,49]. Therefore, future mHealth applications should adopt a more inclusive, couple-centered approach that addresses the reproductive and psychosocial needs of both partners.

The limited use of scientific references in infertility-related mobile applications raises concerns about the scientific basis of the health information provided [39]. In particular, overly definitive predictions regarding ovulation and the likelihood of pregnancy, as well as diet and lifestyle recommendations that are not adequately supported by evidence, may expose users to misleading health information [19,33,39]. Similar concerns apply to unverified claims regarding vitamins or herbal supplements and to treatment recommendations made without individualized clinical evaluation. Moglia et al. reported that many menstrual cycle applications did not meet the accuracy criteria and that even among those considered accurate, the use of scientific literature and involvement of healthcare professionals were limited [19]. Zwingerman et al. similarly identified inaccuracies in the content and tools provided by fertility and menstrual tracking applications [39]. Clay et al., in a systematic qualitative review of 29 studies, reported that application-based ovulation predictions may not align with clinical assessments or ovulation tests and that some application recommendations may conflict with those provided by healthcare professionals [33]. Taken together, these findings underscore the importance of transparent, evidence-informed health information and clear distinctions between digital guidance and individualized clinical assessment.

One potential risk is the use of fertility applications as substitutes for professional healthcare [50]. Evidence that this directly delays clinical evaluation or treatment remains limited [42]. However, a review by Clay et al. reported that some users relied on fertility tracking tools as substitutes for professional health services rather than as complementary tools [33]. This possibility warrants clinical attention, particularly in situations that require timely evaluation, such as advanced maternal age, diminished ovarian reserve, endometriosis, or severe male-factor infertility [50]. Fertility applications should therefore be designed as supportive tools rather than alternatives to professional evaluation and should direct users to appropriate healthcare services when necessary.

Another issue to consider is that application predictions and commercial claims may create unrealistic expectations. Users may develop a false sense of security if they perceive fertility predictions as definitive or interpret application data without professional guidance. Hohmann-Marriott et al. reported that healthcare professionals were concerned about the accuracy of application-based ovulation estimates and that users might be falsely reassured about their fertility [51]. Given the psychological burden of infertility, applications should avoid conveying certainty regarding pregnancy or treatment success and should clearly state the limitations of their predictions.

Finally, the processing of sensitive reproductive health data, such as menstrual cycle information, sexual activity, and pregnancy plans, by fertility applications raises important privacy and data security concerns [52,53]. The literature has reported inadequate informed consent, data sharing with third parties, and limited transparency regarding the use of personal data. Alfawzan et al. evaluated 23 women’s health applications and found that all collected personal health data, 61% allowed location tracking, and 87% shared data with third parties; however, only 57% informed users about data security [52]. Zadushlivy et al. also highlighted privacy risks in reproductive health applications, including IP address tracking, data sharing with third parties, and potential data misuse [53]. These findings suggest that scientific reliability, appropriate clinical guidance, and data security should be considered alongside technical and content-related characteristics when evaluating infertility applications.

The findings of this study should also be interpreted in the context of other frameworks used to evaluate mobile health applications. MARS evaluates applications based on dimensions such as engagement, functionality, aesthetics, and information quality, whereas ORCHA addresses broader digital health quality dimensions such as data privacy, professional and clinical assurance, and usability [14,15,16]. In contrast, the adapted APPLICATIONS Scoring System used in this study evaluates the scope of physiological, psychological, social, and spiritual care content specific to infertility, in addition to technical and usability features. These differences are important when interpreting the mean score of 13.96 out of 29 obtained in this study. This score reflects the scope of holistic infertility care content and the evaluated technical and usability features; it should not be interpreted as an indicator of clinical quality, information accuracy, data security, or regulatory compliance. Because the adapted APPLICATIONS Scoring System, MARS, and ORCHA have different evaluation domains and scoring structures, their scores should not be compared numerically [15,16]. Instead, these frameworks evaluate different, complementary dimensions of mobile health application quality.

Studies using MARS in reproductive health also demonstrate that application quality can vary across dimensions [15]. Zhang et al. evaluated 11 applications related to male reproductive health and reported an average MARS score of 2.7 out of 5; functionality received the highest score, whereas content related to male fertility remained limited [54]. Similarly, Tran et al. evaluated 11 applications for individuals undergoing ART and reported an average MARS score of 3.63 out of 5, with only one intervention supported by published research [55]. Zych et al. evaluated 1402 digital health applications using the ORCHA Baseline Review and reported that although 29.6% of applications performed well in other areas, they underperformed in professional and clinical assurance. User ratings also did not always align with professional/clinical assurance and data privacy [56]. Taken together, the findings of these studies and the present study suggest that technical and usability characteristics alone do not establish comprehensive infertility-specific content or clinical reliability [55,56]. The various dimensions of application quality should therefore be evaluated separately.

### 4.1. Data Privacy, Security, and Regulatory Considerations

The adapted APPLICATIONS Scoring System used in this study did not systematically evaluate data privacy, cybersecurity, or regulatory compliance. These dimensions therefore require separate assessment when interpreting the findings presented here.

Under the European Union’s General Data Protection Regulation (GDPR), information related to reproductive health and sexual life is considered special-category personal data, and the processing of such data is subject to stricter protection requirements [57]. In contrast, some commercial health applications offered directly to consumers in the United States do not fall within the scope of the Health Insurance Portability and Accountability Act (HIPAA) [58]. This situation raises privacy concerns about how sensitive data collected through fertility applications are used and whether such data are shared with third parties for advertising or marketing purposes.

For this reason, when evaluating fertility applications, it is not sufficient to examine only whether a privacy policy or legal disclaimer exists. Future evaluations should also consider what data applications collect, how these data are used, whether they are shared with third parties, and what control users have over their data [57,58]. Therefore, when evaluating or recommending infertility applications, healthcare professionals should consider not only technical usability and content, but also data privacy, security, and compliance with applicable regulations. These factors should be integral considerations in application development and evaluation processes.

### 4.2. Strengths and Limitations

This study has several strengths. First, systematic application store searches conducted at five time points between 2021 and 2025 enabled the repeated identification and screening of potentially eligible applications, while the final evaluation was based on versions available in December 2025. Second, systematic searches were conducted on the Apple App Store and Google Play Store using bilingual (English and Turkish) search terms that included both medical and common end-user terminology, thereby broadening the scope of the search across both major application stores. Third, the adapted APPLICATIONS Scoring System expanded the assessment to include an infertility-specific holistic care component, encompassing the physiological, psychological, social, and spiritual dimensions, in addition to technical and usability characteristics. Finally, two researchers evaluated the applications independently, and inter-rater reliability was analyzed to assess consistency in the evaluation process.

This study has several limitations. First, the evaluation included only applications available in English and Turkish; applications in other languages were excluded. Additionally, application availability and application-store search results can vary by country, region, and search algorithm. Therefore, the findings may not be fully generalizable to different geographic settings or application-store environments. Applications requiring restricted institutional or clinic-specific access were also excluded; therefore, the findings do not represent applications requiring such access. Second, the researchers conducted the evaluation using predetermined criteria. Although predefined scoring criteria were used, some evaluator subjectivity likely remained, particularly in the subjective assessment criteria. The study did not directly assess user experiences, satisfaction, or the perceptions of individuals experiencing infertility. Third, the study evaluated descriptive application features and the comprehensiveness of application content but did not assess clinical effectiveness, safety, diagnostic accuracy, or patient-level outcomes. Therefore, higher adapted APPLICATIONS scores or broader holistic content coverage should not be interpreted as evidence of clinical validity or effectiveness. Although inter-rater reliability was excellent, the 29-point combined score is based on an adapted, study-specific framework rather than a psychometrically validated instrument. Future studies should further evaluate its validity and reliability, including quantitative content-validity assessment and evaluation using independent mHealth datasets. Furthermore, this study did not systematically evaluate data privacy, cybersecurity, or regulatory compliance. Finally, because of the dynamic nature of the mobile application market, the availability, features, and content of applications may have changed during the 2021–2025 search period; therefore, the findings reflect the versions available at the final evaluation in December 2025.

## 5. Conclusions

In the increasingly digital healthcare environment, mobile applications are widely used to support infertility management and conception planning. However, the findings of the present study suggest that these applications should adopt a more holistic approach that considers the psychological, social, and spiritual aspects of infertility alongside physiological aspects. Future applications should be designed using a multidisciplinary, user-centered approach involving healthcare professionals from relevant disciplines and individuals experiencing infertility. Their content should be based on current scientific evidence and clinical guidelines. Applications should integrate psychological support, evidence-based coping strategies, and mechanisms for referral to professional support when necessary. Predominantly woman-centered approaches should also be expanded to include male reproductive health and address the needs of both partners, including shared coping processes. Before applications are recommended by healthcare professionals, they should undergo appropriate evaluation of clinical accuracy, safety, and clinical effectiveness. Data privacy, security, and compliance with relevant regulations should also be integral considerations in application development and evaluation. In conclusion, infertility-related mobile applications should not replace professional care but should serve as digital resources that support comprehensive, evidence-based infertility care.

## Figures and Tables

**Table 3:** Adapted APPLICATIONS scores of infertility-related mobile applications (*n* = 79).

Application Name	Holistic Care Comprehensiveness (0–16)				Technical and Usability Component (0–13)													Total (0–29)
Application Name	Ph	Ps	So	Sp	P1	P2	L	I1	C	A	T	I2	O1	O2	O3	N	S	Total
IVFMind	0	0	0	4	1	1	0	1	0	1	0	0	1	1	0	0	0	10
Glow-Fertility & Ovulation	4	4	4	4	1	1	1	1	0	1	1	1	1	1	1	1	1	28
My Fertility Track & Monitor	4	0	0	0	1	1	0	0	1	1	1	0	1	0	1	1	1	13
Fertility Diet	4	0	0	0	1	1	1	1	0	0	1	0	1	0	1	1	1	13
Fertility Circle: Support Hub	4	4	4	4	1	1	1	1	0	1	0	1	1	1	1	1	1	27
ReUnite Fertility App	4	0	0	0	1	1	0	1	0	1	0	1	1	1	1	1	1	14
Fertility Zone	4	0	4	0	1	1	1	1	0	1	1	1	1	0	1	1	1	19
My Fertility Forums	4	0	4	0	1	1	1	1	0	1	1	1	1	0	1	1	0	18
The Fertility App- IVF & IUI	4	0	4	0	1	1	0	1	0	1	0	1	1	0	1	1	1	17
Female Fertility & Pregnancy H	0	0	0	4	1	1	0	0	0	1	0	0	1	0	0	1	1	10
Ovia Fertility & Cycle Tracker	4	4	4	4	1	1	1	0	0	1	0	1	1	1	1	1	1	26
Fertility View-Fertility and IVF support	4	0	0	0	1	1	0	1	0	0	0	0	1	0	0	0	0	8
Modern Fertility Cycle Tracker	4	0	4	0	1	1	1	0	0	1	0	0	1	0	1	1	1	16
Fertility Astrology	0	0	0	4	0	1	0	1	0	1	0	1	0	0	0	1	0	9
Enhanced Fertility	4	4	4	4	1	1	1	1	0	0	1	1	1	1	1	1	1	27
Kindara: Fertility Tracker	4	0	4	0	1	1	0	0	0	1	0	1	1	0	1	1	1	16
SMP Fertility Rx	4	0	0	0	1	1	0	0	0	1	0	1	1	1	1	1	1	13
Fertility Edge	4	0	0	0	1	1	0	1	0	1	0	0	0	0	0	0	0	8
ExSeed: Sperm & Fertility App	4	0	4	0	1	1	1	1	0	0	0	1	1	0	1	1	1	17
Fertility Drug Calculator: FDC	4	0	0	0	1	1	0	0	0	1	0	1	0	0	1	0	0	9
Posy Fertility	4	0	4	0	0	0	0	0	0	1	0	0	1	0	1	1	1	13
1in4	4	4	4	0	1	1	1	1	0	1	0	1	1	0	1	1	1	22
Tilly: Fertility & IVF Support	4	4	4	4	0	0	0	1	0	1	0	1	1	1	1	1	1	24
Get Pregnant Faster	0	0	4	4	1	1	0	0	0	1	0	1	1	1	1	1	1	17
Evoke Fertility Meditations	0	0	0	4	1	1	0	1	0	1	0	1	1	0	1	1	1	13
Obie: Fertility, Cycle Tracker	4	0	0	0	1	1	1	1	0	0	1	0	1	0	1	1	1	13
Parla	4	0	0	4	1	1	0	1	0	1	0	0	1	0	1	1	1	16
TwoPlus Fertility	0	0	0	4	1	1	0	1	0	1	0	0	1	0	1	1	0	11
Fertility Tracker—Meal Plans	4	0	0	0	1	1	0	0	0	1	0	0	1	0	0	1	1	10
Fertile Affirmations	0	0	0	4	0	1	0	0	0	0	0	1	1	0	1	1	0	9
Embie: IVF & IUI Tracker	4	0	4	0	1	1	1	0	0	1	1	1	1	1	1	1	1	19
Kindbody: Fertility Care	4	0	4	0	1	1	0	1	0	1	0	0	1	0	1	1	1	16
Naula: Your IVF Simplified	4	0	0	0	1	1	0	1	0	1	0	0	1	0	0	0	0	9
IVF Relax	0	0	0	4	1	1	0	0	0	1	0	0	1	0	1	1	1	11
IVF Positivity	0	0	0	4	1	1	0	0	1	0	0	0	1	0	0	0	1	9
Bonzun IVF	4	0	4	0	1	1	0	0	0	1	0	1	1	0	1	1	1	16
Mindful IVF	0	0	0	4	1	1	0	0	0	1	0	1	1	0	1	0	1	11
The Bub Hub	0	0	4	0	1	1	0	1	0	1	0	0	0	0	0	0	0	8
Expectful: Wellness for Moms	4	0	0	4	1	1	0	0	0	1	0	1	1	0	1	0	1	15
Leeaf	4	0	4	0	1	1	0	1	0	1	0	1	1	0	1	1	1	17
ReceptivaDx	4	0	0	0	1	1	0	1	0	1	0	1	1	1	0	1	0	12
Donor Nexus	4	0	0	0	1	1	0	1	0	0	0	0	1	0	1	0	0	9
VaricoHealth	4	0	0	0	1	1	0	1	1	1	0	1	0	0	0	0	0	10
Artificial Insemination—In Vitro Fertilization	4	0	0	0	1	1	0	1	0	1	1	0	1	0	0	1	0	11
Fertility Yoga	4	0	0	4	1	1	0	1	1	0	0	0	1	0	0	1	0	14
IVF Treatment—In Vitro Fertilization Treatment	4	0	0	0	1	1	0	0	1	0	0	0	1	0	0	0	0	8
Female Infertility	4	0	0	4	1	1	1	1	1	1	1	0	1	0	0	1	1	18
Fertility Diet Guide	4	0	0	0	1	1	0	1	0	1	0	0	1	0	1	1	1	12
PCOS Diet 7 Day Meal Plan	4	0	0	0	1	1	0	0	0	0	0	0	1	0	0	0	0	7
Food and Infertility	4	0	0	0	1	1	1	1	1	0	0	0	1	0	0	1	0	11
Female Fertility Protocols Natural Pregnancy Boost	4	0	0	4	1	1	0	1	0	0	0	0	1	1	1	1	1	16
Conceive Fast Yoga—Ovulation	4	0	0	4	1	1	0	0	0	0	0	0	1	1	1	1	1	15
Regimen: ED Guided Program	4	0	0	4	1	1	1	0	0	1	0	0	1	0	0	1	0	14
PCOS Tracker	4	0	0	0	1	1	1	1	0	1	0	1	1	0	1	1	1	14
Uvi: PCOS Diet & Fitness Plans	4	0	4	0	1	1	1	0	0	0	0	1	1	0	1	1	1	16
PCOS Diet & Period Tracker	4	0	0	4	1	1	1	1	0	1	0	0	1	0	0	0	1	15
Fertility Treatment	4	0	0	0	1	1	1	1	1	0	0	0	1	0	0	1	1	12
PCOS Support	4	0	4	0	1	1	1	1	0	1	0	1	1	0	1	1	1	18
PCOS & PCOD Home Remedies Diet Help Healthy Foods	4	0	0	0	1	1	0	1	1	0	0	0	1	0	0	1	0	10
FertilityNow	4	4	4	4	1	1	1	0	0	1	0	0	1	0	1	1	1	24
Sperm Count Advice	4	4	0	4	1	1	1	0	1	0	0	0	1	0	1	1	1	20
Infertility and its Management	4	0	0	0	1	1	1	0	1	0	0	0	1	0	0	1	0	10
IVF Treatment Guide	4	0	0	0	1	1	0	1	0	1	0	0	1	0	0	0	0	9
Baby4You	0	0	4	0	1	1	0	0	0	0	0	1	1	0	0	1	0	9
Female Reproductive Infections	4	0	0	0	1	1	0	1	1	1	0	0	0	0	0	0	0	9
Infertility Cure Get Pregnant	4	4	0	0	1	1	1	1	1	0	1	0	1	0	0	1	1	17
Hidden Secrets of Infertility	4	0	0	0	1	1	1	1	0	0	0	0	1	0	0	1	0	10
Female Fertility	4	0	0	4	1	1	1	1	1	1	1	0	1	0	0	1	1	18
Infertility Solutions	4	0	0	0	1	1	0	1	1	0	0	0	0	0	0	1	0	9
Women’s Health Problems & Solutions	4	0	0	4	1	1	1	1	1	1	0	0	1	0	0	1	0	16
Infertility	4	4	0	0	1	1	0	1	1	1	0	1	0	0	0	1	0	15
Fertility Prayer	0	0	0	4	1	1	0	1	0	0	1	0	1	0	0	1	0	10
PCOS Guide—Fight PCOS Naturally	4	0	4	0	1	1	1	1	0	1	0	0	1	0	0	1	1	16
12 Yoga Poses for 200+ Diseases	0	0	0	4	1	1	0	1	1	0	0	0	1	0	0	0	0	9
Understanding Ovulation & Fertility	4	0	0	0	1	1	0	1	1	0	0	0	1	0	0	1	0	10
Infertility Guide	4	0	0	0	1	1	1	1	0	0	0	0	1	1	0	1	0	11
Mindful IVF: Meditate & Relax	4	4	0	4	0	0	1	0	0	1	0	1	1	1	1	1	1	20
AskFertility	4	0	0	0	1	1	1	0	0	1	1	1	1	0	0	0	0	11
Juno Men’s Fertility Tracker	4	0	4	0	0	0	0	0	0	1	0	1	1	0	0	0	0	11
**Total** ***n*****%**	65 82.3%	11 13.9%	25 31.6%	30 38.0%	73 92.4%	75 94.9%	31 39.2%	50 63.3%	19 24.1%	52 65.8%	14 17.7%	33 41.8%	71 89.9%	15 19.0%	41 51.9%	60 75.9%	45 57.0%	
**Mean ± SD**	6.63 ± 3.50				7.33 ± 2.07													13.96 ± 4.95
**Range (min–max)**	4–16				3–12													7–28

Abbreviations: Ph, physiological; Ps, psychological; So, social; Sp, spiritual; P1, price; P2, paid subscription; L, literature used; I1, in-app purchases; C, connectivity; A, advertisements; T, text search field; I2, inter-platform compatibility; O1, images/figures; O2, videos; O3, special features; N, navigation ease; S, subjective presentation; SD, standard deviation. Ph, Ps, So, and Sp: 0 or 4; technical and usability criteria: 0 or 1.

**Table 4:** Comparison of adapted APPLICATIONS scores by application characteristics.

Application Characteristics	*n* (%)	Adapted APPLICATIONS Score Median (IQR)	Test Statistic/*p*
**Platform**			***H*** **= 12.438;** ***p*****= 0.002**
iOS only	28 (35.4)	11 (7)	
Android only	18 (22.8)	11 (5)	
Both	33 (41.8)	16 (8)	
**Price**			***U*** **= 203.50;** ***p*****= 0.773**
Free	73 (92.4)	13 (7)	
Paid	6 (7.6)	12 (9)	
**Developer type**			***H*** **= 62.645;** ***p*****< 0.001**
Healthcare professional	26 (32.9)	18 (7)	
Software company	25 (31.6)	13 (4)	
Unknown developer	28 (35.5)	9 (1)	
**Target audience**			***H*** **= 44.251;** ***p*****< 0.001**
Couples	38 (48.1)	16 (4)	
Women	35 (44.3)	10 (2)	
Men	6 (7.6)	11 (4)	

Note: IQR = interquartile range; H = Kruskal–Wallis H test; U = Mann–Whitney U test. Pairwise comparisons were adjusted using the Bonferroni correction.

## Data Availability

The data supporting the findings of this study are presented in the article.
